# National trends of pre-hypertension and hypertension among Iranian adolescents across urban and rural areas (2007–2011)

**DOI:** 10.1186/s13293-019-0230-1

**Published:** 2019-03-29

**Authors:** Parisa Amiri, Golnaz Vahedi-Notash, Parisa Naseri, Davood Khalili, Seyed Saeed Hashemi Nazari, Yadollah Mehrabi, Ali Reza Mahdavi Hazaveh, Fereidoun Azizi, Farzad Hadaegh

**Affiliations:** 1 Research Center for Social Determinants of Health, Research Institute for Endocrine Sciences, Shahid Beheshti University of Medical Sciences, Tehran, Iran; 2 Department of Biostatistics, School of Paramedical Sciences, Shahid Beheshti University of Medical Sciences, Tehran, Iran; 3 Prevention of Metabolic Disorders Research Center, Research Institute for Endocrine Sciences, Shahid Beheshti University of Medical Sciences, Tehran, Iran; 4 Safety Promotion and Injury Prevention Research Center, Department of Epidemiology, School of Public Health and Safety, Shahid Beheshti University of Medical Sciences, Tehran, Iran; 5 Department of Epidemiology, School of Public Health, Shahid Beheshti University of Medical Sciences, Tehran, Iran; 6 Center for Non-communicable Diseases Control, Ministry of Health and Medical Education, Tehran, Iran; 7 Endocrine Research Center, Research Institute for Endocrine Sciences, Shahid Beheshti University of Medical Sciences, Tehran, Iran

**Keywords:** Hypertension, Pre-hypertension, Adolescents, Urban, Rural

## Abstract

**Background:**

The current nationwide study, for the first time, aimed to assess and compare the trend of pre-hypertension and hypertension among urban and rural adolescents in Iran.

**Methods:**

This study has been conducted in the framework of the National Surveys of Risk Factors for Non-Communicable Diseases. To estimate pre-hypertension and hypertension prevalence among 9715 adolescents, aged 15–19 years, data collected in four repeated cross-sectional surveys (2007–2011) has been used. The prevalence trends of pre-hypertension and hypertension were examined across urban and rural areas of Iran. To calculate the adjusted prevalence ratios (PRs) of pre-hypertension and hypertension over cycles across area of residence and genders, a complex sample survey and multinomial logistic analysis were performed.

**Results:**

Using the definition of pre-hypertension and hypertension presented by the seventh Joint National Committee (JNC-VII) for adolescents, after adjusting for confounders, the prevalence of pre-hypertension changed in both urban (boys:28.96% to 29.24% and girls:18.33% to 20.06%) and rural (boys 31.58% to 32.05% and girls 22.25% to 24.13%) areas over the study duration. Non-significant rising prevalence of hypertension was also observed in boys and girls of both regions (urban 12.76% to 15.04% and 8.02% to 9.06%; rural 9.95% to 11.79% and 10.35% to 11.60%, for boys and girls respectively). The adjusted prevalence ratios (PRs) of pre-hypertension (2.16; 95% CI 1.68–2.79 and 1.92, 95% CI 1.57–2.34, in urban and rural, respectively) and hypertension (2.40; 95% CI 1.65–3.51 and 1.82, 95% CI 1.36–2.45, in urban and rural, respectively) were higher in boys than girls. Comparing the adjusted PRs of pre-hypertension and hypertension in urban versus rural areas, in both genders, showed higher PRs of pre-hypertension in rural girls (1.33, 95% CI 1.01–1.75).

**Conclusion:**

The current results showed high constant trends of pre-hypertension and hypertension in Iranian boys and girls, residing in both urban and rural areas.

## Background

Hypertension plays a pivotal role in predisposing adult population to cardiovascular diseases (CVDs), in the Middle East and North Africa (MENA)) regions [1, 2]. Accordingly, a recent WHO report indicates a considerable prevalence of hypertension in the Eastern Mediterranean region (EMR), ranging from 14.7% to 26.4% in different countries of this region [3]. Findings of a large national survey showed that 42.7% of Iranian adults suffer from high blood pressure including pre-hypertension and hypertension, according to the seventh report of the Joint National Committee on Prevention, Detection, Evaluation, and Treatment of High Blood Pressure (JNC-VII) [4]. It is well documented that hypertension beginning in childhood and adolescence is a predictor of high blood pressure in adulthood, contributing to early development of CVD disorders [5]. Although the latest pooled prevalence of hypertension among Iranian children and adolescents was estimated to be 8.9%, slightly higher in boys than girls (10.3% vs 9.1%), and has a wide range (4.2 to 24.2%) in different ages and residential areas of Iran [6, 7]. Hypertension is reported to be the most common comorbidity of overweight/obesity, showing an increasing trend in Iranian children as a probable consequence of their lifestyle changes through recent decades [8, 9].

Urbanization, a process through which changes in industries and economy cause expansion of cities and alter the way populations live, can significantly affect many aspects of a population’s lifestyle, which could predispose them to CVD risk factors, including hypertension [10–12]. Existing data show the significant influence of urbanization and its consequences on the patterns of childhood physical activity, sedentary behaviors, and diet, in both developed and developing countries [13, 14]. While a number of studies revealed unhealthy lifestyles as being more prevalent among urban children [15], further evidence indicated some sort of homogeneity between urban and rural lifestyles through recent decades [14]; which can be attributed to the fast rising trend of modernization in particular development of amenities and greater access to communication technologies [16].

Iran has also been witnessing an era of urbanization over the past three decades. The most recent census in Iran showed that almost 74% of the Iranian population live in urban areas, i.e., just 25% of Iranians are residents of rural areas. Previous statistics of populations living in urban areas were 71 and 68%, according to censuses conducted in 2011 and 2006, respectively, indicating that although Iran is experiencing a slow, but steady, increase in the urban population, a significant proportion of Iranians still resides in rural areas (https://nnt.sci.org.ir/sites/nnt/SitePages/report_90/population_report.aspx). Despite the expansion of urbanization and the alarming rate of hypertension in non-Western countries, less attention has been paid to comparing trends of hypertension and its comparison in urban and rural adolescents; for example, one study investigating urban–rural disparity in blood pressure among Chinese children between 1985 and 2010, showed higher blood pressure levels in rural children than their urban counterparts, which could not be explained by body mass index (BMI) status [17]. Data from Iran shows differences in mean systolic and diastolic blood pressure (SBP and DBP, respectively) between adults in urban and rural populations, in and around the city of Isfahan in central Iran [18]. However, there is no study comparing the trend of childhood pre-hypertension and hypertension between urban and rural residential areas of the country. Given this gap in evidence, the present study aimed to assess the trend of pre-hypertension and hypertension in pediatric populations of urban versus rural areas in Iran.

## Methods

### Study population

This study has been conducted within the framework of the national Surveillance of Risk Factors of Non-Communicable Diseases (SuRFNCD), using guidelines of the stepwise approach to NCD risk factor surveillance of the World Health Organization [19]. The SuRFNCD, which consists of several cross-sectional studies, was initiated in 2004 and repeated almost annually until 2011. Briefly, using a multistage random cluster sampling method, data for a nationally represented sample of urban and rural populations of all provinces of Iran has been assessed through each survey. Data were collected by trained nurses during household interviews. Details of the SuRFNCD have been published previously [20].

Adolescent boys and girls, aged 15–19 years, whose SBP and DBP were above the mean ± 3SD, were considered as outliers and were excluded from the study; hence, those who participated in the 2007 (*n* = 2936), 2008 (*n* = 2988), 2009 (*n* = 2862), and 2011 (*n* = 929) surveys, entered the study. The surveys received ethical approval of the Center for Disease Control of Iran. Data recorded on age, sex, residential area, physical activity, BMI, SBP, and DBP were analyzed.

### Measurements

After at least 10 min of resting in the sitting position two measurements of blood pressure (BP) were obtained within 5 minutes of each other, from the right arm of participants; by trained health-staff using an appropriate sized cuff [20]. Blood pressure was measured manually with a calibrated Omron M7 sphygmomanometer (HEM-780-E), and the mean of the two abovementioned measurements was considered as the participant’s BP.

BMI was calculated as the weight in kilograms divided by the square of height in meters.

Physical activity was obtained using the global physical activity questionnaire (GPAQ), a standardized questionnaire developed by WHO, as a physical activity surveillance instrument. The translation of the second version of GPAQ was used for the assessment of physical activity [21]. Another questionnaire was used to collect demographic data, including occupation, sex, age, province, and area.

### Definition of terms

For adolescents, aged 15–17 years, normal blood pressure was defined as systolic blood pressures (SBP) and diastolic blood pressures (DBP) < 90th percentile, and pre-hypertension was defined as SBP or DBP ≥ 90th but < 95th percentile, or BP levels ≥ 120/80 mmHg, considering the participant’s age-sex-height. In addition, hypertension was defined as SBP and/or DBP ≥ 95th percentile. For adolescents, aged 18–19 years, pre-hypertension was defined as either SBP of > 120 but < 140 mmHg or DBP of > 80 but < 90 mmHg; hypertension was defined as SBP ≥ 140 mmHg and DBP ≥ 90 mmHg, or taking antihypertensive medication [22] . Based on the GPAQ analysis framework, physical activity was defined at the three following levels: (1) *High*: vigorous-intensity activity, on at least 3 days a week, achieving a minimum of at least 1500 MET minutes per week, or 7 days of any combination of walking and moderate- or vigorous-intensity activities, achieving a minimum of at least 3000 MET-minutes per week; (2) *Moderate*: three or more days of vigorous-intensity activity of at least 20 min per day, or five or more days of moderate-intensity activity (including walking) of at least 30 min per day, or five or more days of any combination of walking, moderate- or vigorous intensity activities, achieving a minimum of at least 600 MET-minutes per week; and (3) *Low*: not meeting any of the abovementioned criteria [21].

### Statistical analysis

Complex sample survey analysis was performed to obtain representative estimates of the Iranian population, in 2007, 2008, 2009, and 2011. Weights used for this analysis were generated for sex and area of residence (rural/urban), according to the population of Iran (national census, 2011).

Data from 2007 to 2011 SuRFNCD were pooled. Characteristics of participants were summarized as mean ± standard error and frequencies (percentages), for continuous and categorical variables, respectively; the unadjusted and adjusted prevalence rates and their 95% confidence intervals were estimated.

Sex-stratified prevalence rate of pre-hypertension and hypertension over SuRFNCD cycles across area of residence were computed by taking the predicted marginal from the logistic regression model. Differences in linear trends in the prevalence of pre-hypertension and hypertension over SuRFNCD cycles across the area of residence were examined by modeling interaction terms (e.g., area × SuRFNCD cycle) for boys and girls, separately.

Multinomial logistic was used to test for temporal trends of pre-hypertension and hypertension prevalence across the 5 SuRFNCD cycles. In models, blood pressure levels were considered as dependent categorical variable and time was included as a categorical variable as follows: 0 for 2007, 1 for 2008, 2 for 2009, and 4 for 2011. The time variable was used to assess temporal trends.

Adjusted prevalence ratios (PRs) for sex (girls as reference) were shown in two different models; model 1, adjusted for year (1^a^), and model 2 further adjusted for physical activity and BMI (2^a^). A similar approach was applied for calculating adjusted PRs for the residential area (urban as reference) (model 1^b^ and 2^b^, respectively). PRs and 95% CIs were calculated for all models.

All analyses were performed using STATA version 14.0, and *P* values < .05 were considered statistically significant.

## Results

A total of 9715 (5257 boys; 4458 girls) urban and rural adolescents, aged 15–19 years, who participated in SuRFNCD between 2007 and 2011, were recruited in the current analysis.

Socio-demographic and anthropometric characteristics, based on the urban and rural areas, have been presented in Table 1. To provide more details of the study participants, a sex-specific table has also been considered as a supplementary document (Table 4 in Appendix).

**Table 1:** Area-specific characteristics of adolescents, aged 15–19 years: SuRFNCD 2007–2011

Year	Urban				Rural			
	2007 (*n* = 1718)	2008 (*n* = 1819)	2009 (*n* = 1550)	2011 (*n* = 615)	2007 (*n* = 1218)	2008 (*n* = 1169)	2009 (*n* = 1312)	2011 (*n* = 314)
Age (years)	17.60 ± 0.03	17.59 ± 0.03	17.59 ± 0.04	17.73 ± 0.06	17.55 ± 0.04	17.56 ± 0.04	17.61 ± 0.04	17.62 ± 0.08
Sex (%)								
Girls	751 (43.7)	811 (44.6)	716 (46.2)	332 (54)	551 (45.2)	528 (45.2)	603 (46)	166 (52.9)
Boys	967 (56.3)	1008 (55.4)	834 (53.8)	283 (46)	667 (54.8)	641 (54.8)	709 (54)	148 (47.1)
Physical activity (% ± SE)								
High	38.39 ± 1.02	33.50 ± 0.99	32.98 ± 1.07	33.35 ± 1.76	49.25 ± 1.27	48.99 ± 1.27	49.45 ± 1.21	36.96 ± 2.49
Moderate	29.80 ± 1.10	25.27 ± 1.01	28.81 ± 1.14	22.63 ± 1.66	24.50 ± 1.22	19.99 ± 1.16	22.26 ± 1.15	21.84 ± 2.30
Low	31.80 ± 1.06	41.21 ± 1.06	38.20 ± 1.16	44.01 ± 1.80	26.3 ± 1.18	31.01 ± 1.25	28.27 ± 1.13	41.18 ± 2.50
BMI (kg/m^2^)	21.73 ± 0.08	21.80 ± 0.08	21.80 ± 0.09	21.86 ± 0.16	21.18 ± 0.08	21.30 ± 0.09	21.41 ± 0.09	21.21 ± 0.21
SBP (mmHg)	114.09 ± 0.28	112.83 ± 0.28	113.69 ± 0.28	115.56 ± 0.48	115.57 ± 0.34	115.03 ± 0.35	115.07 ± 0.33	116.73 ± 0.63
DBP (mmHg)	72.77 ± 0.22	72.77 ± 0.21	73.58 ± 0.22	72.09 ± 0.39	71.59 ± 0.26	72.10 ± 0.27	71.79 ± 0.25	70.60 ± 0.49

Age, physical activity, BMI, SBP, and DBP values are mean ± SEM. Variables (except sex) are weighted according to sex

**Fig. 1:**
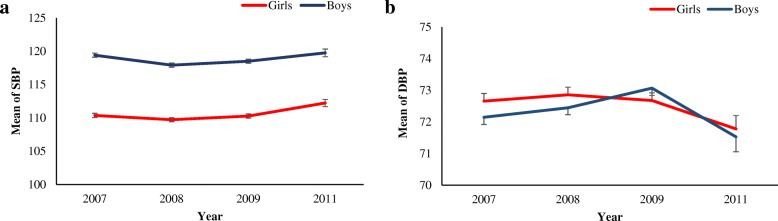
**a** Gender-specific trend of systolic blood pressure (SBP) in adolescents aged 15–19 years: SuRFNCD 2007–2011. **b** Gender-specific trend of diastolic blood pressure (DBP) in adolescents aged 15–19 years: SuRFNCD 2007–2011. Error bars show standard errors of mean

**Fig. 2:**
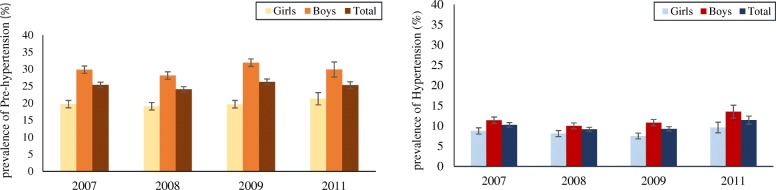
Prevalence of pre-hypertension and hypertension based on JNC-VII guideline among girls, boys, and the total adolescent population in SuRFNCD 2007–2011

The average SBP was changed from 119.41 to 119.76 and 110.37 to 112.24 in SuRFNCD 2007–2011 for boys and girls; corresponding values for DBP changed from 72.15 to 71.53 and 72.66 to 71.78, respectively (Fig. 1). For more clarification, average SBP and DBP based on gender/area groups are illustrated in Figure 4 in Appendix. In addition, using the JNC-VII guideline and regardless of residential area, prevalence of both pre-hypertension and hypertension in SuRFNCD 2007–2011 for the whole population and separately for genders is illustrated in Fig. 2. Accordingly, the prevalence of pre-hypertension and hypertension changed from 25.34 and 10.25 in 2007 to 25.29 and 11.41% in 2011 among the whole population.

**Fig. 3:**
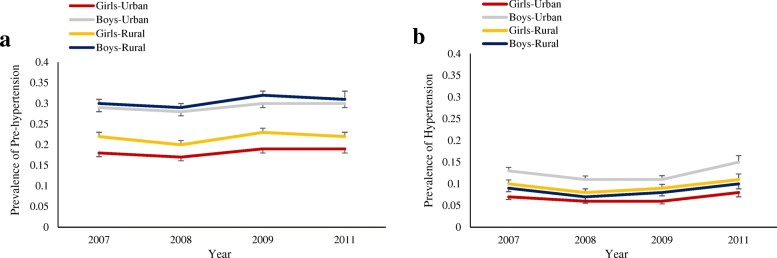
**a** Gender/area-specific trend for unadjusted prevalence of pre-hypertension in Iranian adolescents, aged 15–19 years based on JNC-VII guideline: SuRFNCD 2007–2011. *P*-interaction for girls-area = 0.59 and for boys-area = 0.31. **b** Gender/area-specific trend for unadjusted prevalence of hypertension in Iranian adolescents, aged 15–19 years, based on JNC-VII guideline: SuRFNCD 2007–2011. *P*-interaction for girls-area = 0.95 and for boys-area = 0.13. Error bars show standard errors of marginal effects

**Fig. 4:**
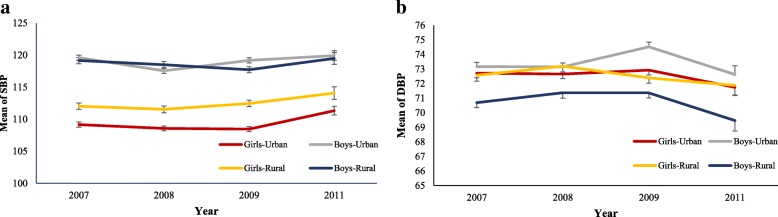
**a** Gender/area-specific trend of systolic blood pressure (SBP) in adolescents aged 15–19 years: SuRFNCD 2007–2011. **b** Gender/area-specific trend of diastolic blood pressure (DBP) in adolescents aged 15–19 years: SuRFNCD 2007–2011. Error bars show standard errors of mean

The prevalence of pre-hypertension and hypertension for different gender/area categories, based on the four national surveys mentioned, is illustrated in Fig. 3a and b. In both genders, the interactions of area and SuRFNCD cycles were not significant, either for pre-hypertension (*P*-interaction = 0.31 and 0.59 for boys and girls, respectively) or hypertension status (*P*-interaction = 0.13 and 0.95 for boys and girls, respectively). In addition, unadjusted prevalence of pre-hypertension and hypertension is shown in Table 5 in Appendix of the manuscript.

The highest prevalence of pre-hypertension for boys (urban 31.61% and rural 34.34%) and girls (urban 20.06% and rural 24.13%), was documented in 2009 and 2011, respectively. However, in both genders and areas, the highest prevalence of hypertension was observed in the last survey, conducted in 2011 (urban boys 15.04% and rural boys 11.79%; urban girls 9.06% and rural girls 11.60%). The adjusted prevalence of pre-hypertension and hypertension showed a constant trend in both genders and residential areas. In urban areas, prevalence of pre-hypertension in adolescents changed from 28.96 to 29.24% and 18.33 to 20.06% in boys and girls, respectively; and in rural adolescents it rose from 31.58% to 32.05% and 22.25% to 24.13%, in boys and girls, respectively. A slightly rising prevalence of hypertension was also observed in boys and girls of both regions, (urban 12.76 to 15.04% and 8.02 to 9.06%; rural 9.95 to 11.79% and 10.35 to 11.60%, for boys and girls, respectively) (Table 2).

**Table 2:** Adjusted prevalence and trend of pre-hypertension and hypertension based on JNC-VII guideline in adolescents aged 15–19: SuRFNCD 2007–2011

Year	2007	2008	2009	2011	*P* for trend
Girls					
Urban					
Pre-hypertension	18.33 (15.91–20.75)	17.71 (15.36–20.06)	18.04 (15.55–20.52)	20.06 (16.41–23.71)	0.540
Hypertension	8.02 (6.31–9.72)	6.98 (5.49–8.46)	6.56 (5.04–8.08)	9.06 (6.44–11.69)	0.510
Rural					
Pre-hypertension	22.25 (19.37–25.13)	21.63 (18.76–24.50)	22.04 (19.15–24.92)	24.13 (19.74–28.52)	0.279
Hypertension	10.35 (8.19–12.52)	9.07 (6.99–11.14)	8.53 (6.57–10.48)	11.60 (8.29–14.91)	0.425
Boys					
Urban					
Pre-hypertension	28.96 (26.39–31.54)	27.28 (24.87–29.69)	31.61 (28.83–34.39)	29.24 (24.83–33.66)	0.937
Hypertension	12.76 (10.82–14.71)	10.81 (9.12–12.50)	12.27 (10.26–14.28)	15.04 (11.26–18.82)	0.099
Rural					
Pre-hypertension	31.58 (28.36–34.80)	26.65 (26.59–32.71)	34.34 (31.10–37.58)	32.05 (27.03–37.07)	0.182
Hypertension	9.95 (7.89–12.01)	8.39 (6.59–10.20)	9.53 (7.61–11.44)	11.79 (8.69–14.89)	0.502

Prevalence rates were adjusted for physical activity and BMI

The gender/area-specific PRs of pre-hypertension and hypertension are presented in Table 3. The year-adjusted PRs of pre-hypertension and hypertension for boys were higher than girls, in both residential areas (model 1^a^). Similarly, after further adjustment for physical activity and BMI, the PRs of pre-hypertension (2.16, 95% CI 1.68–2.79 and 1.92, 95% CI 1.57–2.34—urban and rural, respectively) and hypertension (2.40, 95% CI 1.65–3.51 and 1.82, 95% CI 1.36–2.45—urban and rural, respectively) were higher in boys than girls (model 2^a^). Comparing PRs of pre-hypertension and hypertension in urban boys and girls with their rural counterparts, the year-adjusted results showed higher PR of pre-hypertension for rural girls than their urban counterparts, and lower PR of hypertension in rural compared to urban boys (model 1^b^). However, after further adjustment for physical activity and BMI, only the rural girls showed higher PR of pre-hypertension than their urban counterparts (1.33, 95% CI 1.01–1.75) (model 2^b^).

**Table 3:** Prevalence ratios of pre-hypertension and hypertension based on JNC-VII guideline in adolescents aged 15–19 years: SuRFNCD 2007–2011

		Urban		Rural	
		Pre-hypertension	Hypertension	Pre-hypertension	Hypertension
Model 1^a^	Girls	Reference	Reference	Reference	Reference
	Boys	2.06(1.65–2.59)	2.20(1.58–3.07)	1.88(1.58–2.24)	1.73(1.33–2.23)
Model 2^a^	Girls	Reference	Reference	Reference	Reference
	Boys	2.16(1.68–2.79)	2.40(1.65–3.51)	1.92(1.57–2.34)	1.82(1.36–2.45)
		Girls		Boys	
		Pre-hypertension	Hypertension	Pre-hypertension	Hypertension
Model 1^b^	Urban	Reference	Reference	Reference	Reference
	Rural	1.32(1.01–1.72)	1.42(0.97–2.08)	0.99(0.78–1.26)	0.65(0.46–0.92)
Model 2^b^	Urban	Reference	Reference	Reference	Reference
	Rural	1.33(1.01–1.75)	1.41(0.96–2.09)	1.08(0.85–1.38)	0.77(0.53–1.11)

Model 1^a^: adjusted model for year

Model 1^b^: adjusted model for year

Model 2^a^: adjusted model for year, physical activity, and BMI

Model 2^b^: adjusted model for year, physical activity, and BMI

Reference: Normal group

**Fig. 5:**
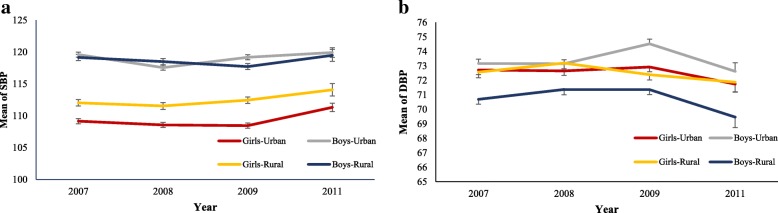
Prevalence of pre-hypertension and hypertension based on AHA guideline among girls, boys, and the total adolescent population in SuRFNCD 2007–2011

As a sensitivity analysis, using the new American Heart Association (AHA) guideline (pre-hypertension 120/≤ 80 to 129/≤ 80 mmHg and hypertension ≥ 130/≥ 80 mmHg) [23] regardless of residential area, prevalence of both pre-hypertension and hypertension in SuRFNCD 2007–2011 for the whole population and in boys and girls separately is illustrated in Figure 5 in Appendix. Compared with the JNC-VII criteria, applying AHA cut points leads to a lower prevalence rate of pre-hypertension, but a significant increase in prevalence of hypertension in SuRFNCD 2007–2011. Using the AHA guideline, the prevalence of pre-hypertension and hypertension in the whole population changed from 14.23 to 29.18% and from 16.79 to 27.66% in the 2007 and 2011 surveys, respectively.

**Fig. 6:**
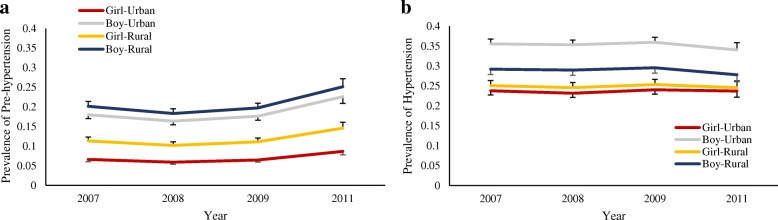
**a** Gender/area-specific trend for unadjusted prevalence of pre-hypertension in Iranian adolescents, aged 15–19 years based on AHA guideline: SuRFNCD 2007–2011. *P*-interaction for girls/area = 0.39 and for boys/area = 0.26. **b** Gender/area-specific trend for unadjusted prevalence of hypertension in Iranian adolescents, aged 15–19 years, based on AHA guideline: SuRFNCD 2007–2011. *P*-interaction for girls-area = 0.99 and for boys-area = 0.49. Error bars show standard errors of marginal effects

Moreover, the unadjusted prevalence and trend of pre-hypertension and hypertension using the new AHA guideline, based on residential area/sex groups, are shown in Table 6 and Figure 6 in Appendix.

## Discussion

To the best of our knowledge, this is the first study in the MENA region and the third worldwide assessing the trend of high blood pressure among adolescents. The results of this study indicate a high constant prevalence of pre-hypertension and hypertension in boys and girls residing in both urban and rural areas, throughout the study period. For both areas, an overall higher prevalence of pre-hypertension and hypertension was observed in boys than girls; except for pre-hypertension that was significantly more prevalent in rural girls compared to their urban counterparts, the prevalence of pre-hypertension and hypertension was geographically independent.

The current findings regarding the considerable prevalence of pre-hypertension and hypertension, among Iranian adolescents, are consistent with some previous regional reports, indicating high blood pressure to be a nationwide problem among this age group in Iran [6, 7]. However, due to age variations, the comparison of the current results with other nationwide studies focused on Iranian school-aged children would be difficult [24, 25]. Our results for all participants, regardless of gender and residential area, indicate the prevalence of pre-hypertension to be 24 and 25% for the first (2007) and the last (2011) examinations, respectively; the corresponding values for hypertension were 10 and 11%, respectively (Fig. 2). More gender-specific results show a higher prevalence of pre-hypertension and hypertension in Iranian adolescent boys than girls. Comparing current results with existing evidence from other countries, data from NHANES in 2003–2006 [26] and 2011–2013 [27] show the prevalence of elevated BP, including both pre-hypertension and hypertension, among US adolescents aged 13–17 years, to be 2.6% and 1.3%, respectively. In addition, data from China in 2004 indicate the prevalence of pre-hypertension and hypertension to be 9.5% and 21.0% among adolescents aged 13–17 years [28]. Another study (2010) revealed prevalence of 17–25% among 15–17-year-old Chinese adolescents suffering from hypertension [29].

Factors affecting hypertension and pre-hypertension in pediatric populations are multiple. Data from NHANES [30], and another study in China [31], indicate that the major determinant of the recent increase in the prevalence of childhood hypertension is a concomitant increase in childhood obesity. However, adjustments for BMI in these studies suggested that other influencing factors are involved. In the current study, BMI adjusted trends of pre-hypertension and hypertension indicate a weight independent trend of elevated blood pressure in Iranian adolescents as well. According to protocol of the SuRFNCD, unfortunately, for participants aged < 25 years, no blood sampling has been performed [19], so information on fasting blood sugar and lipid profiles in this age group is not available for the current analysis. However, another national study conducted between 2009 and 2010 among 5940 Iranian adolescents, aged 10–18, reported the prevalence of 12.8% and 18.45% for high FBS among boys and girls, respectively; corresponding values for high TG (TG ≥ 130 mg/dl) were 14.8% and 13.8%, respectively [32]. This considerable prevalence of cardio-metabolic risk factors among Iranian children might be attributed to nutrition transition in Iran and the preference of Iranian families for Western eating patterns through the last decades [33] which could be independent of their weight status [34]. In this regard, more evidence shows 73.8% of Iranian school-aged children consumed hydrogenated solid fat, and about 80% of them add table salt to their food [25].

The current study shows similar constant trends of pre-hypertension and hypertension in both urban and rural Iranian adolescents, through 2007–2011. Previous data comparing high blood pressure and its main determinants among Iranian children, residing in urban and rural areas, are sparse. Looking through international data indicates that the urban/rural disparity for high blood pressure during early years of life is controversial and might vary between countries. While some studies revealed cardio-metabolic risk factors to be geographically independent [35, 36], other evidence suggest a significant difference in the prevalence of high blood pressure between urban and rural children [37, 38], without any specific explanation for the cause of this disparity [39].

The current similar trend of early high blood pressure in urban and rural areas of Iran could be explained by a major lifestyle transition, especially the increased dominance of Western food patterns among Iranian families over the last decades, resulting in 2 fold burden of nutritional disorders among Iranian children, particularly in rural areas [40]. Consistently, a recent nationwide study revealed that although Iranian children in rural areas were more physically active, they had less healthy dietary habits than their urban counterparts, especially in regions with a low socio-economic status (SES) [41], findings in agreement with those of several previous studies, which documented high physical activity [42–45], high consumption of traditional/salty meals [2, 46], and high prevalence of obesity among rural adolescents [46].

Although the current results indicate an overall higher probability of pre-hypertension and hypertension in boys than girls, rural girls compared to their counterparts in urban areas were at higher risk for pre-hypertension. Despite the lack of accurate behavioral assessments in the current study, data from another nationwide study in Iran indicated that, compared to boys, girls, in regions with different SES, were less physically active [41]; this gender difference in lifestyle habits could be explained by the different cultural conditions and social opportunities for boys and girls, which has also been reported by other studies in Iran [47] and a majority of other Muslim communities as well [48, 49].

As for strengths, this is the first nationwide gender/area-specific report on the trend of pre-hypertension and hypertension among Iranian adolescents, preformed over four repeated cross-sectional surveys. Regarding limitations, data on dietary status of the participants was not available; hence, we could not consider this important confounder in data analysis. In addition, following widespread migration from rural to urban areas in Iran, during the recent decades, we were unable to consider marginalization and its influence on lifestyle patterns and diseases in this study. Thus, future research should include sub-urban children, as a third comparison group, considering their behavioral, cultural, and environmental characteristics, which could affect their blood pressure status.

## Conclusions

Overall, the current results show constant high trends of pre-hypertension and hypertension in Iranian boys and girls, residing in both urban and rural areas. Moreover, boys were more likely to be affected for both pre-hypertension and hypertension than girls, and rural girls showed higher prevalence ratio of pre-hypertension than their urban counterparts. Future research targeted at genetic, behavioral, and socio-environmental determinants underlying and influencing these constant high trends of elevated blood pressure among adolescent should be considered in both urban and rural areas of the country.

## Appendix

**Table 4:** Sex-specific characteristics of adolescents, aged 15–19 years: SuRFNCD 2007–2011

Year	Girls				Boys			
	2007 (*n* = 1302)	2008 (*n* = 1339)	2009 (*n* = 1319)	2011 (*n* = 498)	2007 (*n* = 1634)	2008 (*n* = 1649)	2009 (*n* = 1543)	2011 (*n* = 431)
Age (years)	17.67 ± 0.03	17.69 ± 0.03	17.57 ± 0.04	17.69 ± 0.06	17.50 ± 0.03	17.46 ± 0.03	17.61 ± 0.03	17.70 ± 0.07
Residential area (%)								
Urban	751 (57.7)	811 (60. 6)	716 (54.3)	332 (66.7)	967 (59.2)	1008 (61.1)	834 (54.1)	283 (65.7)
Rural	551 (42.3)	528 (39.4)	603 (45.7)	166 (33.3)	667 (40.8)	641 (38.9)	709 (45.9)	148 (34.3)
Physical activity (% ± SE)								
High	19.66 ± 1.08	19.21 ± 1.05	18.37 ± 1.05	16.07 ± 1.63	63.56 ± 1.19	57.29 ± 1.16	57.70 ± 1.27	52.50 ± 2.36
Moderate	35.68 ± 1.35	27.97 ± 1.22	30.91 ± 1.31	23.90 ± 1.83	20.70 ± 1.02	19.28 ± 0.97	22.60 ± 1.10	20.90 ± 1.98
Low	44.65 ± 1.37	52.80 ± 1.34	50.71 ± 1.39	60.01 ± 2.09	15.73 ± 0.90	23.42 ± 0.98	19.68 ± 1.04	26.58 ± 2.05
BMI(kg/m^2^)	21.78 ± 0.09	21.92 ± 0.10	21.94 ± 0.11	22.03 ± 0.18	21.33 ± 0.08	21.37 ± 0.08	21.40 ± 0.09	21.27 ± 0.17
SBP(mmHg)	109.89 ± 0.31	109.35 ± 0.31	109.63 ± 0.31	112.21 ± 0.52	119.21 ± 0.30	117.63 ± 0.30	118.63 ± 0.30	119.61 ± 0.56
DBP(mmHg)	72.58 ± 0.25	72.71 ± 0.24	72.71 ± 0.25	71.74 ± 0.41	72.20 ± 0.23	72.40 ± 0.22	73.29 ± 0.24	71.49 ± 0.45

Age, physical activity, BMI, SBP, and DBP values are mean ± SEM

Variables (except residential area) are weighted according to residential area

**Table 5:** Unadjusted prevalence and trend of pre-hypertension and hypertension based on JNC-VII in Iranian adolescents: SuRFNCD 2007–2011

Year	2007	2008	2009	2011	*P* for trend
Girls					
Urban					
Pre-hypertension	18.18 (15.90–20.47)	17.66 (15.44–19.89)	18.04 (15.72–20.36)	19.92 (16.42–23.42)	0.624
Hypertension	7.86 (6.23–9.49)	7.04 (5.63–8.46)	6.56 (5.08–8.04)	8.78 (6.30–11.27)	0.500
Rural					
Pre-hypertension	21.99 (19.14–24.85)	21.47 (18.64–24.29)	21.93 (19.11–24.76)	23.89 (19.59–28.18)	0.322
Hypertension	10.27 (8.14–12.39)	9.24 (7.20–11.28)	8.62 (6.68–10.55)	11.37 (8.22–14.53)	0.595
Boys					
Urban					
Pre-hypertension	29.40 (26.89–31.90)	27.57 (25.18–29.97)	32.26 (29.53–34.99)	29.20 (24.79–33.60)	0.936
Hypertension	12.94 (11.08–14.79)	11.25 (9.53–12.97)	12.38 (10.43–14.34)	15.18 (11.47–18.89)	0.108
Rural					
Pre-hypertension	30.75 (27.78–33.71)	28.66 (25.77–31.56)	33.67 (20.62–36.72)	30.78 (25.91–35.66)	0.244
Hypertension	8.87 (7.13–10.62)	7.67 (6.08–9.26)	8.48 (6.77–10.18)	10.50 (7.80–13.19)	0.390

**Table 6:** Unadjusted prevalence and trend of pre-hypertension and hypertension based on AHA guideline in Iranian adolescents: SuRFNCD 2007–2011

Year	2007	2008	2009	2011	*P* for trend
Girls					
Urban					
Pre-hypertension	6.10 (4.72–7.48)	5.97 (4.64–7.29)	5.80 (4.52–7.09)	9.82 (7.31–12.32)	0.18
Hypertension	23.59 (21.04–26.15)	23.86 (21.34–26.38)	22.95 (20.39–25.50)	24.28 (20.42–28.15)	0.61
Rural					
Pre-hypertension	10.51 (8.32–12.71)	10.29 (8.16–12.42)	10.04 (7.90–12.17)	16.42 (12.22–20.61)	0.06
Hypertension	24.98 (22.04–27.92)	25.27 (22.28–28.27)	24.37 (21.50–27.25)	24.93 (20.72–29.14)	0.69
Boys					
Urban					
Pre-hypertension	18.59 (16.46–20.73)	16.32 (14.34–18.31)	18.35 (16.11–20.60)	21.33 (17.48–25.17)	0.66
Hypertension	35.71 (33.08–38.34)	34.61 (32.07–37.16)	37.01 (34.20–39.82)	33.51 (28.96–38.07)	0.78
Rural					
Pre-hypertension	20.80 (18.18–23.42)	18.21 (15.76–20.67)	20.60 (17.97–23.23)	23.70 (19.22–28.17)	0.12
Hypertension	29.32 (26.49–32.15)	28.35 (25.55–31.16)	30.50 (27.63–33.38)	27.34 (23.11–31.56)	0.51

## Abbreviations

BMI: Body mass index
BP: Blood pressure
CVDs: Cardiovascular diseases
DBP: Diastolic blood pressure
EMR: Eastern Mediterranean region
GPAQ: Global physical activity questionnaire
PRs: Prevalence ratios
SBP: Systolic blood pressure
SES: Socio-economic status
