# Correction to: National trends of pre-hypertension and hypertension among Iranian adolescents across urban and rural areas (2007–2011)

**DOI:** 10.1186/s13293-019-0234-x

**Published:** 2019-04-15

**Authors:** Parisa Amiri, Golnaz Vahedi-Notash, Parisa Naseri, Davood Khalili, Seyed Saeed Hashemi Nazari, Yadollah Mehrabi, Ali Reza Mahdavi Hazaveh, Fereidoun Azizi, Farzad Hadaegh

**Affiliations:** 1 Research Center for Social Determinants of Health, Research Institute for Endocrine Sciences, Shahid Beheshti University of Medical Sciences, Tehran, Iran; 2 Department of Biostatistics, School of Paramedical Sciences, Shahid Beheshti University of Medical Sciences, Tehran, Iran; 3 Prevention of Metabolic Disorders Research Center, Research Institute for Endocrine Sciences, Shahid Beheshti University of Medical Sciences, Tehran, Iran; 4 Safety Promotion and Injury Prevention Research Center, Department of Epidemiology, School of Public Health and Safety, Shahid Beheshti University of Medical Sciences, Tehran, Iran; 5 Department of Epidemiology, School of Public Health, Shahid Beheshti University of Medical Sciences, Tehran, Iran; 6 Center for Non-communicable Diseases Control, Ministry of Health and Medical Education, Tehran, Iran; 7 Endocrine Research Center, Research Institute for Endocrine Sciences, Shahid Beheshti University of Medical Sciences, Tehran, Iran

**Correction to: Biology of Sex Differences**

**https://doi.org/10.1186/s13293-019-0230-1**

Following publication of the original article [1], it was brought to our attention that Fig. 4 and Fig. 5 erroneously have the same graph published. The corrected Fig. 5 is given below.

**Fig. 5:**
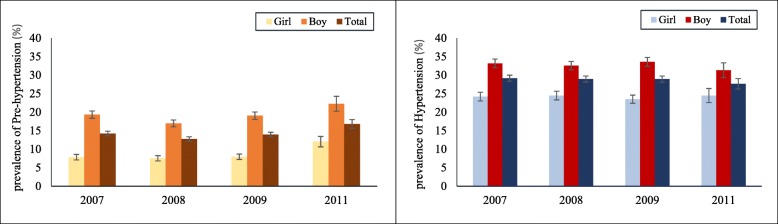
Prevalence of pre-hypertension and hypertension based on AHA guideline among girls, boys, and the total adolescent population in SuRFNCD 2007–2011

The original article [1] has been corrected. We apologise for any inconvenience caused.
